# The Belgian health examination survey: objectives, design and methods

**DOI:** 10.1186/s13690-020-00428-9

**Published:** 2020-06-03

**Authors:** Diem Nguyen, Pauline Hautekiet, Finaba Berete, Elise Braekman, Rana Charafeddine, Stefaan Demarest, Sabine Drieskens, Lydia Gisle, Lize Hermans, Jean Tafforeau, Johan Van der Heyden

**Affiliations:** 1 Department of Epidemiology and public health, Sciensano, Juliette Wytsmanstraat 14, 1050 Brussels, Belgium; 2 Department of Chemical and physical health risks, Sciensano, Brussels, Belgium; 3 Centre for Environmental Sciences, University of Hasselt, Hasselt, Belgium; 4 Unit of Epidemiology and Social Medicine, University of Antwerp, Antwerp, Belgium

**Keywords:** Health examination survey, Belgium, Survey methodology

## Abstract

**Background:**

In 2018 the first Belgian Health Examination Survey (BELHES) took place. The target group included all Belgian residents aged 18 years and older. The BELHES was organized as a second stage of the sixth Belgian Health Interview Survey (BHIS). This paper describes the study design, recruitment method and the methodological choices that were made in the BELHES.

**Methods:**

After a pilot period during the first quarter of the BHIS fieldwork, eligible BHIS participants were invited to participate in the BELHES until a predefined number (*n* = 1100) was reached. To obtain the required sample size, 4918 eligible BHIS participants had to be contacted. Data were collected at the participant’s home by trained nurses. The data collection included: 1) a short set of questions through a face-to-face interview, 2) a clinical examination consisting of the measurement of height, weight, waist circumference, blood pressure and for people aged 50 years and older handgrip strength and 3) a collection of blood and urine samples. The BELHES followed as much as possible the guidelines provided in the framework of the European Health Examination Survey (EHES) initiative. Finally 1184 individuals participated in the BELHES, resulting in a participation rate of 24.1%. Results for all the core BELHES measurements were obtained for more than 90% of the participants.

**Conclusion:**

It is feasible to organize a health examination survey as a second stage of the BHIS. The first successfully organized BELHES provides useful information to support Belgian health decision-makers and health professionals. As the BELHES followed EHES recommendations to a large extent, the results can be compared with those from similar surveys in other EU (European Union) member states.

## Background

Non-communicable diseases such as cardiovascular diseases and diabetes contribute substantially to disability, morbidity and mortality all over the world. Information about the prevalence of non-communicable diseases and the occurrence of associated risk factors is required in order to develop and evaluate health policies to prevent and control these diseases [1].

Both health interview surveys (HISs) and health examination surveys (HESs) have an important role in obtaining this type of information. HISs provide data on the health status, health behaviors, medical care consumption and social and demographic characteristics of the general population through interviews and self-administered questionnaires. HESs focus on objective information collected through clinical examinations and analysis of biological samples which cannot be collected in a HIS.

HESs have been organized in many countries and are often combined with a HIS. One of the most famous HESs is the National Health and Nutrition Examination Survey (NHANES) in the US, which has been conducted periodically since the beginning of the sixties. The NHANES consists of two parts: a questionnaire at the participant’s home and a standardized health examination in an equipped mobile examination center [2]. Many European countries have also organized a HES in the past decades. Between 2007 and 2017 national HESs were conducted in 14 EU countries [3]. A lot of expertise is available in Finland and the United Kingdom (UK). Finnish HESs include the FINRISK Surveys from 1972 to 2012, the Mini-Finland Survey from 1978 to 1980, the Health 2000/2011 Surveys and the FinHealth 2017 [4]. The Health Survey for England (HSE) is an annual cross-sectional HES in the non-institutionalized English general population since 1991 [5]. Recent HESs in countries neighboring Belgium are the German Health Interview and Examination Survey for Adults (DEGS) [6], “Nederland De Maat Genomen” in the Netherlands [7], « l’étude de santé sur l’environnement, la biosurveillance, l’activité physique et la nutrition » Esteban [8] in France and the European Health Examination Survey in Luxemburg (EHES-LUX) [9].

Although many countries have HES information, it is often difficult to compare the results due to methodological differences. The European Health Examination Survey (EHES) was an initiative to set up a system of standardized, representative HESs in the EU member states [3]. The core measurements and analyses proposed by EHES (i.e. measurement of height, weight, waste circumference, blood pressure, glycaemia, cholesterolaemia and glycolated haemoglobin) are all related to major risk factors of chronic diseases. EHES developed methodological guidelines to support new initiatives in EU member states starting up a new HES, therefore enhancing comparability in the future [10].

In Belgium, HISs have been conducted since 1997, with intervals between 3 to 5 years [11]. In 2017, it was decided to add a HES component to the Belgian Health Interview Survey (BHIS) 2018. Due to budgetary constraints, the HES was only organized in a subsample of the BHIS participants. The Belgian Health Examination Survey (BELHES) was commissioned by the National Institute of Disability and Health Insurance and organized by the Department Epidemiology and public health of Sciensano, the national public health institute, in collaboration with Statistics Belgium (Statbel) the national office of statistics.

The main objectives of the BELHES 2018 were:

- to test the feasibility of implementing a HES in Belgium in the general adult population;
- to obtain objective information from the general adult Belgian population on the prevalence of important biomedical risk factors for cardiovascular diseases and diabetes: overweight and obesity, hypertension, hypercholesterolaemia and hyperglycaemia;
- to study the association between those risk factors with health status, health determinants and socio-demographic information collected through the BHIS.

The additional objectives were:

- to measure the handgrip strength of the population aged 50 years and older, as part of the assessment of the prevalence of frailty in this population group;
- to measure salt intake and iodine deficiency at population level;
- to validate information on smoking and passive smoking collected in the BHIS through measurement of cotinine and hydroxycotinine in urine;
- to investigate whether outdoor air pollution contributes to health effects by integrating biomarkers of exposure and effect;
- to set up a biobank with biological samples for future research in public health.

The objectives of this paper are:

- to assess the feasibility of implementing in Belgium a HES with limited resources, adhering as much as possible to EHES recommendations;
- to describe the study design, recruitment method and methodological choices that were made;
- to provide information on the participation rate, the obtained sample and the measurements that were carried out.

## Methods

### Study design

The BHIS 2018 was a cross-sectional epidemiologic study in which information was obtained on the health status, health behavior and health consumption of the general Belgian population. Detailed information on the BHIS methodology can be found elsewhere [11]. The BELHES was organized as a second stage of the BHIS 2018.

### Target population and sampling frame

The sampling frame of the BHIS consisted of all households listed in the National Registry (NR). For practical reasons, people living in an institution (prisons, religious communities or cloisters that shelter more than 8 persons, psychiatric institutions,…) were excluded with the exception of older people residing in nursing homes. Information was collected via a computer assisted personal interviewing (CAPI) and a self-administered questionnaire. If the selected person was not able to answer him/herself or was absent for a long time, proxy interviews were conducted. However, no information was available for these persons on smoking, alcohol use, mental health and other modules which were considered as sensitive or too personal to be included in the face-to-face interview.

The target population of the BELHES included all persons with residence in Belgium without any restrictions on nationality. The sampling frame consisted of all persons who participated in the BHIS 2018 except for minors (< 18 years), BHIS participants for whom a proxy interview was conducted and residents of the German Community. Minors were excluded from the target population because the prevalence of cardiovascular risk factors is low in this age group and obtaining informed consent is complex.

### Sampling scheme

BHIS participants were selected through a multistage stratified sampling which is described elsewhere [11]. Due to financial constraints the BELHES sample size had to be restricted to the minimum number that was required to obtain information on the overall prevalence of the main EHES core indicators (elevated blood pressure, serum cholesterol, diabetes and obesity) in the three Belgian regions with a reasonable precision. It was calculated that for an estimated true proportion of 0.5, a desired precision of 0.05 in each of the three regions and a confidence level of 0.95 the total sample size should be 1155 individuals. After negotiation with the commissioners this was rounded to 1100.

The BHIS started in January 2018. During February and March 2018 a BELHES pilot data collection was organized among eligible BHIS participants of 11 municipalities in two regions (Brussels and Wallonia). The fieldwork of the pilot study was carried out by two trained nurses. All procedures in the protocol, starting from the invitation of eligible BHIS participants up to the reception and the validation of all data were tested. At the end of the pilot project an evaluation meeting was held with the field nurses, the laboratory, the HES data manager and the HES project coordinator to assess if adaptations to the protocol were necessary.

On the 1st of April 2018 the actual BELHES survey was launched in the whole country and continued throughout 2018. Based on the anticipated number of BHIS participants being eligible for BELHES participation from April onwards (i.e. after the pilot study), it was estimated that a participation rate of 20 to 25% would be required to achieve the aimed target sample size. As a Dutch study found a HES participation rate as low as 30% [12], it was decided to recruit from the start onwards all eligible BHIS participants and to stop recruiting when the regional quota would be reached. Regional quota had been set to 450, 300 and 350 in respectively the Flemish, Brussels and Walloon region. These quota were based on the regional stratification in the HIS sample.

### Selection of physical measurements and biological samples

In a HES it is of utmost importance that measurements are obtained in a standardized way. In many European countries it has been shown that standardization of physical measurements in a national HES is feasible [13]. In the BELHES it was aimed to profit as much as possible from the experience that was achieved in the framework of the EHES. Therefore EHES core measurements were given priority in the choice of the BELHES measurements. The selection of these measurements was based on public health importance, clear interpretation of results, availability of international standards, easiness to administer, lack of other sources to provide information, costs, ethical acceptability and participant acceptability.

A list of all clinical measurements and biological samples of the BELHES is presented in Table 1.

**Table 1:** Clinical measurements and biological samples in the BELHES

Clinical measurement		Biological samples			
		Blood		Urine	
EHES core	Extra	EHES core	Extra	EHES core	Extra
Blood pressure Height Weight Waist Circumference	Handgrip strength (50+ years)	HbA1C^a^ Glucose Cholesterol	Telomere length Mitochondrial DNA^b^	None	Cotinine Hydroxycotinine Sodium Iodine Creatinine Black carbon PAHs^c^

^a^Glycated haemoglobin

^b^Deoxyribonucleic acid

^c^Polyaromatic hydrocarbons

#### Questionnaires

The EHES guidelines recommend to include questions on household size, gender, age, marital status, socio-economic status (education, occupation, household income), self-reported height and weight, general health, chronic diseases, use of medication and smoking. As these topics were already collected in the BHIS, they were not included in the BELHES questionnaire. A complete list of the modules included in the BHIS 2018 is presented in Additional file 1.

Data linkage between the BHIS and the BELHES data was based on a unique project number assigned to each participant, but for verification purposes information on birth date and gender was included both in the BHIS and the BELHES questionnaire.

The BELHES questionnaire also included questions to assess exclusion criteria for blood collection and questions addressing the circumstances in which measurements were done. If certain measurements could not be performed, the reason had to be recorded.

Medicine consumption may interfere with the assessment of certain clinical measurements such as blood pressure and the outcomes of the biological measurements (cholesterolaemia, glycaemia). Therefore, participants were asked whether they had used medicines for hypertension, high cholesterol and diabetes in the past 2 weeks and specific information was obtained on all medicines that had been taken in the 24 h before the examination.

To embed the measurement of the handgrip strength in a broader perspective, the frailty instrument used in the Survey of Health, Ageing and Retirement (SHARE) [14, 15] was also included in the BELHES questionnaire. This instrument measures the five dimensions of Fried’s frailty phenotype: exhaustion, weight loss, slowness, low activity and weakness. As such, the weakness dimension of Fried’s frailty phenotype was assessed both with the handgrip test [16, 17] and the question used in the SHARE frailty instrument.

#### Clinical measurements

Several clinical measurements were performed in the BELHES (see Table 1). Blood pressure was measured with an electronic tensiometer (type Omron M6) with the participant in sitting position. Three measurements were done with an interval of 1 min. In line with EHES recommendations, the mean of the second and the third measurement was considered as the value reflecting the actual blood pressure.

Since people tend to overreport their height and underreport their weight [18], height and weight measurement is an essential part of a HES, hence also included in the BELHES. People were measured without shoes and with the lightest clothing as possible. The weight was measured to the nearest 0.5 kg using an calibrated electronic scale (type SECA 804). The body height was measured to the nearest 0.1 cm with a portable stadiometer (type SECA 213) following the Frankfort horizontal plane for the head position.

The Body Mass Index (BMI) was calculated to assess the prevalence of overweight and obesity. The BMI, however, is not a good indicator of body fatness and does not provide information on the distribution of abdominal adiposity. Several studies demonstrated that waist circumference is a better predictor of adverse health outcomes than BMI [19–21]. Waist circumference reflects the accumulation of visceral fat and is significantly associated with a higher risk of incident cardiovascular events and type 2 diabetes. A larger waist circumference (men: > 102 cm, women: > 88 cm) predicts higher levels of vascular risk factors, and a higher incidence of vascular events [22]. The waist circumference was measured in light or without clothing to the nearest 0.5 cm with a measuring tape (type Meterex) between the lower rib and the iliac crest.

Grip strength is a simple measure of overall muscle strength and has shown to have health-related prognostic value [23]. In the BELHES, handgrip strength was measured in people aged 50+ with a dynamometer, type Smedley (100 kg). Three measurements were performed [24, 25].

#### Biological samples

In the BELHES, two types of biological samples were collected: blood and urine (see Table 1). Exclusion criteria for blood collection were adopted from the HSE and included pregnancy, clotting disorders, the use of anticoagulant medicines and a history of epileptic fits. Participants had to be fasting for at least 8 h. Blood samples were first of all taken for the EHES core analyses related to cardiovascular risk factors and diabetes: glucose, cholesterol (total and High Density Lipoprotein) and HbA1c. Additionally, an extra blood sample was taken for DNA extraction to determine telomere length and mitochondrial DNA content. These analyses were planned in the framework of a research project in the domain of environmental health. Some additional blood samples were frozen and stored in a biobank for further research in the domain of public health.

Spot urine was collected to determine sodium, iodine, creatinine, cotinine and hydroxycotinine, black carbon load and polyaromatic hydrocarbons (PAHs). The measurement of sodium in spot urine provides information on the sodium intake at population level. It has been well established that excessive dietary salt intake raises blood pressure. Reducing average population salt levels has been identified as a strategy for preventing stroke and other cardiovascular diseases, and the World Health Organization (WHO) aims to reduce population salt intake by 30% by 2025 [26]. The implementation of this strategy requires baseline and serial follow-up measurements of mean population salt intake in order to document the progress towards this goal. Urinary iodine is a well-accepted, cost-efficient and easily obtainable indicator for iodine status at the population level [27]. Although an individual’s urinary iodine concentration could vary daily, or even within the same day, these variations tend to even out within populations, providing a useful measure of the iodine status of the population. Nicotine exposure results into the presence of cotinine and hydroxycotinine in bodily fluids [28]. Urine concentration of cotinine and hydroxycotinine is an indicator of passive and active smoking and provides additional information to the information on self-reported smoking in the BHIS [29]. Black carbon load and PAHs were measured as biomarkers of environmental health exposure. Excessive or reduced fluid intake can substantially alter the concentration of substances in urine. As a standard to detect urinary dilution, creatinine concentrations are required. For this reason also creatinine was analyzed in the urine. Finally a urine sample was frozen and stored in a biobank for further studies in the domain of public health.

### Fieldwork procedures

Health examinations were performed at the participants’ home. Belgium was divided into 27 geographical areas, each covered by one nurse. The geographical areas were defined in such a way that the nurse did not have to drive longer than 1 h to reach the participant’s home. Nurses were recruited through national and regional nursing organizations and received a specific theoretical and practical training.

For the collection, analyses and storage of biological samples, a collaboration was set up with a private laboratory. This lab was selected through a call for tender in which quality criteria where specified in accordance to the EHES guidelines.

**Fig. 1:**
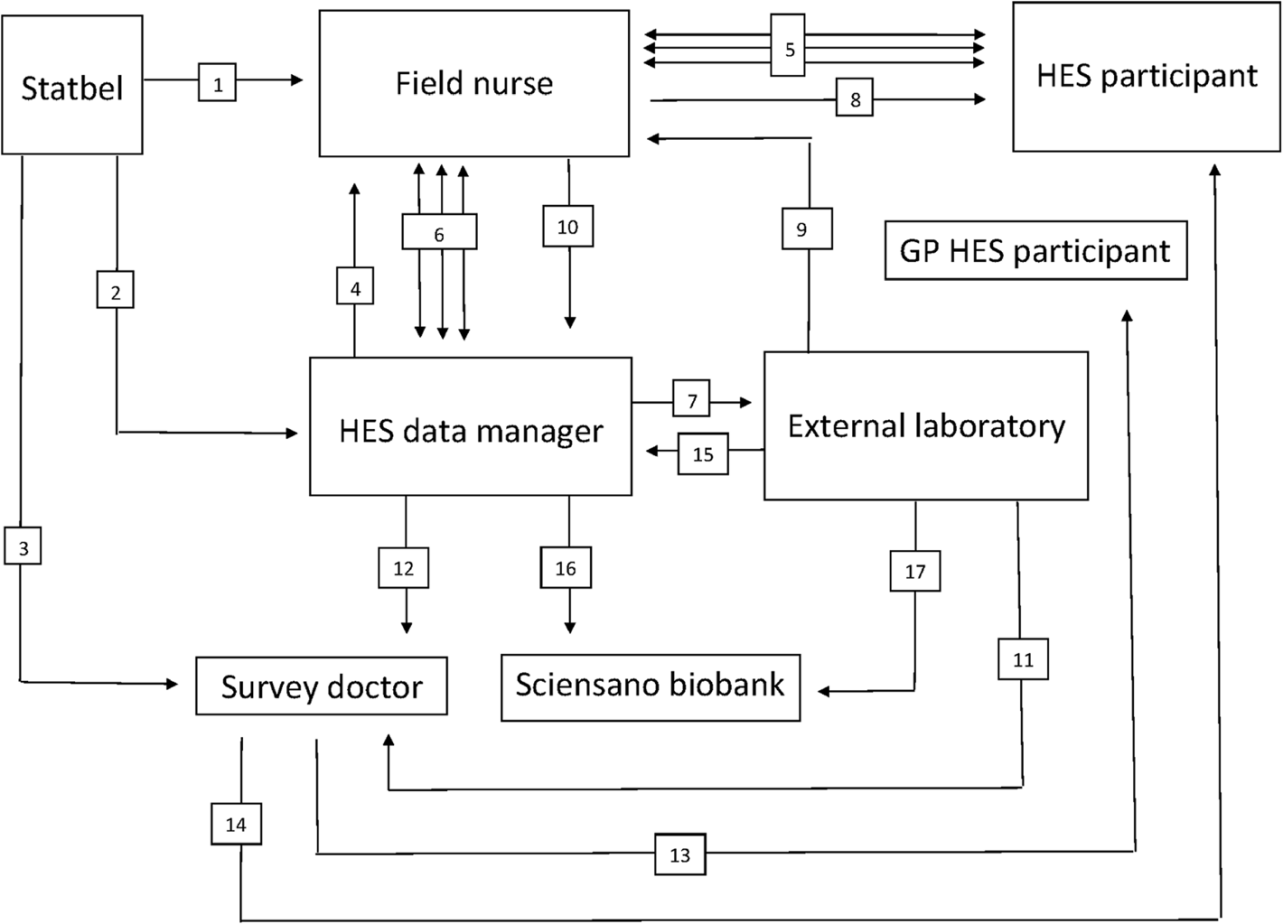
BELHES data collection steps (numbers refer to the steps described under “Fieldwork procedures”)

The data collection was organized in collaboration with Statistics Belgium (Statbel). Figure 1 illustrates the different steps in the data collection and should be viewed in combination with the explanation below.

Eligible BHIS participants were asked whether they were willing to participate in the BELHES. If so, their coordinates (name, address, phone number, e-mail address) were sent to Statbel. On a weekly basis Statbel sent the coordinates and the BELHES identification (ID) code to the nurse in charge of the area where the person lived [1]. The BELHES data manager was informed of the BELHES ID numbers of the participants [2]. The survey doctor received both the BELHES ID and the coordinates of the potential participants [3]. As soon as the data manager received the BELHES ID from Statbel this information was entered on a secured web based form, which was also accessible to the nurses [4]. The data manager had access to the forms of all nurses. Each nurse only had access to his/her own form. After receiving the participants’ coordinates, nurses started contacting people to make an appointment [5]. Information on the status of each respondent was entered and regularly updated via the webform [6], which enabled the data manager to monitor the progress of the fieldwork data collection.

When the nurse made an appointment, this information was entered on the webform [6]. The data manager checked all webforms on a daily basis. The day before the appointment, the data manager informed the laboratory where samples had to be collected the following day [7]. On the day of the appointment, the nurse went to the participant’s home [8] to obtain informed consent, fill in the short questionnaire via CAPI, perform the health examinations and collect the blood and urine samples. The samples were brought to a collection point near the nurses’ home before 12 o’clock, where they were collected by the laboratory in the afternoon to be analysed the same day [9].

The CAPI data were transferred to the data manager via a secured Internet connection [10]. At the level of the laboratory, coded results were sent to the survey doctor through a secured transfer [11]. Urine and blood samples that were not directly analyzed were stored at − 80 °C. BELHES results received by the data manager were also made available to the survey doctor [12]. Based on these data, together with the coded laboratory data and the participant’s coordinates, which the survey doctor received from Statbel, the survey doctor prepared feedback letters for both the participant [13] and his or her general practitioner [14]. At regular times the laboratory gave feedback to the data manager on the received samples [15]. The data manager communicated this information to the Sciensano biobank [16]. Every 1 to 2 months, frozen BELHES samples were transferred from the laboratory to the Sciensano biobank where they were stored at − 80 °C [17].

### Outcome of the study

Eligible BHIS participants were systematically invited to participate in the BELHES until fieldwork parameters indicated that the required sample size was about to be reached. At that moment 4918 eligible BHIS participants had been invited. Since the procedures were not changed after the pilot data collection, the participants of the pilot study were included in the final study sample. Finally 21 people participated in the pilot study and 1163 in the actual survey, leading to a total sample size of 1184 individuals and a global participation rate of 24,1%. Table 2 presents more detailed information on the participation per region.

**Table 2:** National and regional BELHES participation rates

	Belgium	Flanders	Brussels	Wallonia
BHIS 2018 participants (n)	11,611	4296	3099	4216
BHIS 2018 participants eligible for BELHES participation, but not contacted^a^ (n)	391	101	203	87
BHIS 2018 participants eligible and contacted for BELHES participation (n)	4918	1878	1337	1703
BHIS 2018 participants that participated in the BELHES (n)	1184	546	275	363
BHIS 2018 participants that refused to participate in the BELHES (n)	3734	1332	1062	1340
Participation rate (%) ^b^	24.1%	29.1%	20.6%	21.3%

^a^Not contactable after initial agreement or not invited for participation because quota was reached

^b^Participated/participated + refused

The participation rates were respectively 29.1, 20.6 and 21.3% in the Flemish, Brussels and Walloon regions. Participation rates also differed substantially in function of age. Lower participation rates were observed in the age groups 18–24 years (21.5%) and 65+ years (19.3%) than in the age groups of 25–44 years (27.3%) and 45–64 years (25.4%). Another important determinant of BELHES participation was the educational attainment. Participation rates were respectively 16.5% among people with no diploma or only primary education, 17.7% among people with a lower secondary diploma, 24.5% among people with a higher secondary diploma and 26.6% among people with a diploma of higher education.

After adjustment for age and gender, the participation rate was significantly higher in the two higher than in the two lower educational groups (odds ratio 1.26; 95% CI 1.07–1.48).

**Fig. 2:**
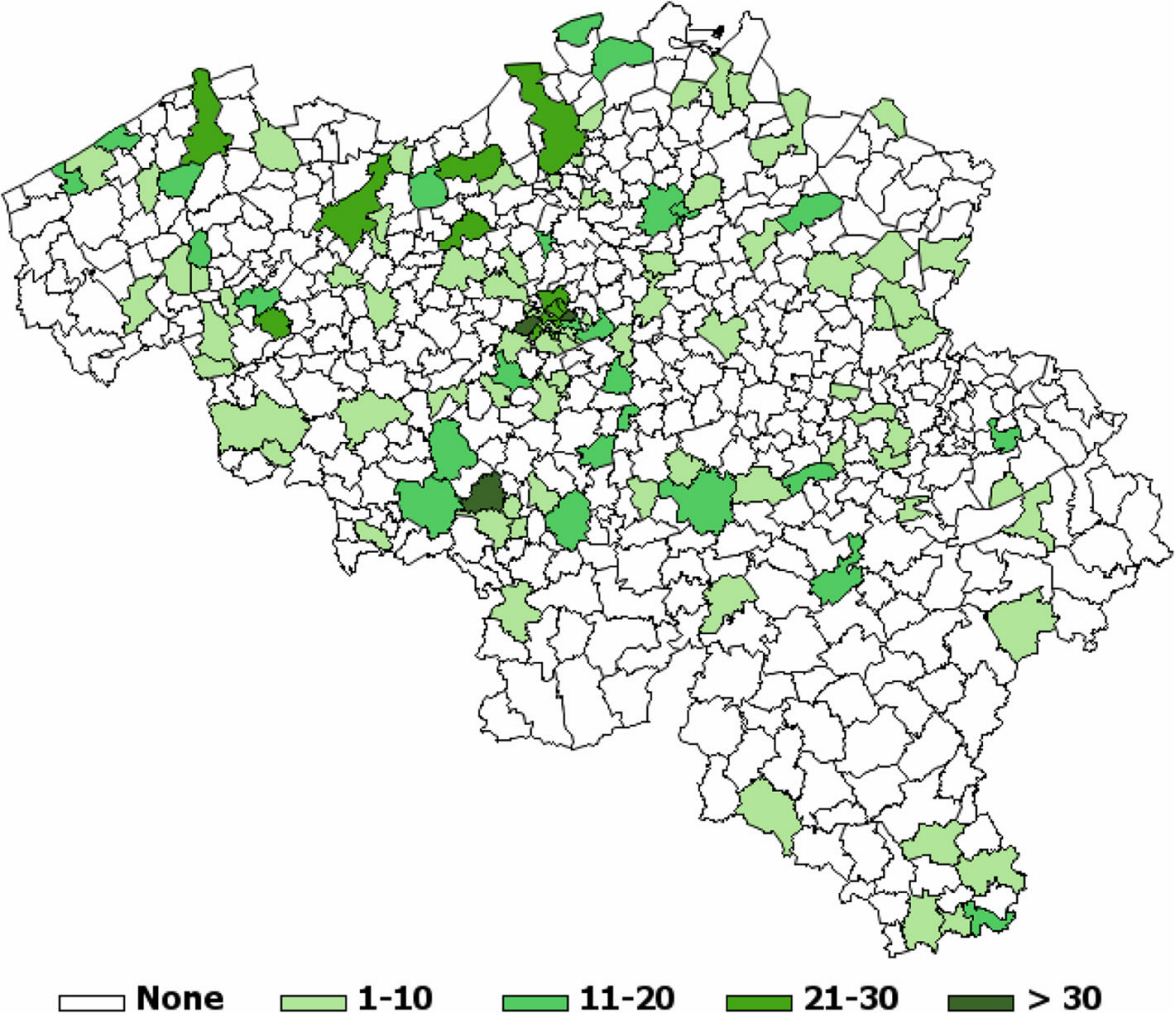
Number of BELHES participants by municipality

The final BELHES sample included residents from 149 of a total of 589 Belgian municipalities, scattered all over Belgium (Fig. 2). The recruitment of BELHES participants ended in December 2018, but health examinations were carried out until February 2019.

**Fig. 3:**
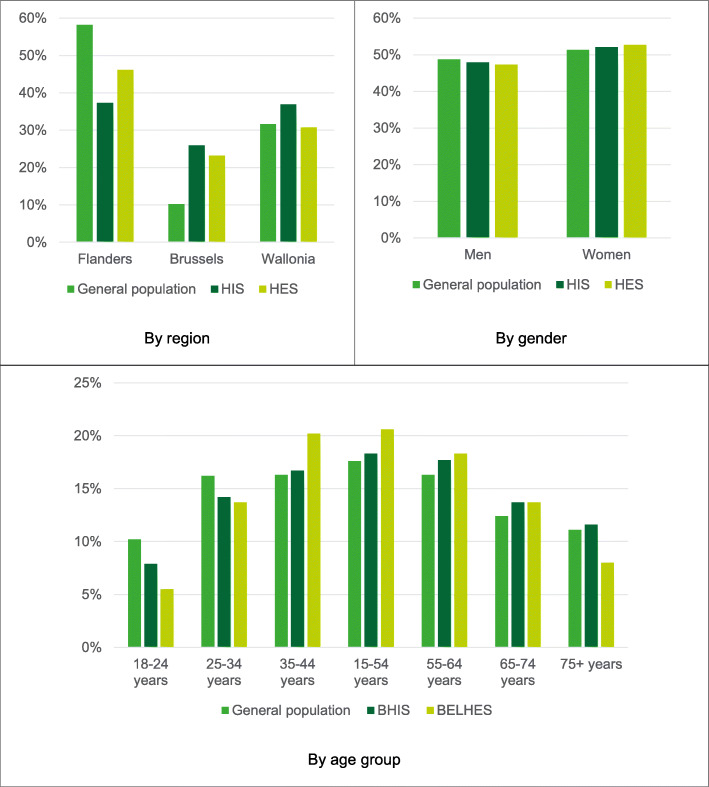
Distributions of the general population aged 18 years and older, the BHIS sample and the BELHES sample by region, gender and age group

Figure 3 presents the distribution of the BELHES sample, the BHIS sample and the general population of 18 years and older by region, gender and age. Compared to the general population, the BELHES sample included a slightly higher proportion of women and substantially less people in the younger (18–24 years, 25–34 years) and the older (75 years and older) age groups. Compared to the BHIS sample, underrepresented age groups in the BELHES were the youngest age group (18–24 years) and the oldest age group (75+ years).

The average duration of the health examination was 23 min. Information on the clinical examination was available for all BELHES participants, but was incomplete for 18 BELHES participants (1.5%), e.g. because no weight or waste circumference was measured if a woman was pregnant or due to a problem that had occurred during the examination, such as a technical problem with the tensiometer.

Blood collection was not possible in 109 BELHES participants (9.2%) due to selective refusal and difficulty in finding a vein. As such, from 90.8% of the participants, all BELHES measurements were obtained.

The timely collection of BELHES samples from all over the country to one central laboratory was challenging from a logistical point of view. However, in 98.2% of the cases blood and urine arrived at the laboratory on the same day. The mean delay between the blood collection and analysis in the laboratory was 7 h and 43 min (median: 7 h and 37 min).

## Discussion

The BELHES is the first national HES organized in Belgium. The study showed that even with limited resources it is feasible to organize a HES as a second stage of the BHIS, which adheres to a great extent to EHES recommendations. The predefined sample size was achieved, and although the participation rate was only 24,1%, the sample distribution by age group and gender resembled well the one of the Belgian general population, except for a slight underrepresentation of people in the age groups 18–24 years and 75 years and over. Clinical measurements and biological samples were obtained from most participants. The participation rate was rather low, but the weighted distribution of the BELHES sample by age, gender and province resembled very well the distribution of those characteristics in the general Belgian adult population.

Previous HES-related activities in Belgium are scarce. However, there is one recent project that needs to be mentioned. Under the auspices of the INTERREG IV A program, 2007–2013, the Nutrition, Environment and Cardiovascular health survey (NESCAV) was organized in three cross border regions including the Grand-Duchy of Luxembourg, Wallonia in Belgium and Lorraine in France. Similar to the HES, an important aim of the NESCAV was the assessment of the cardiovascular health and risk profile of the population [30]. Nevertheless, the BELHES has two important advantages compared to the NESCAV: it has been organized in the whole country and EHES recommendations were followed as much as possible to enhance comparability in a broader European context.

The organization of a HES implies several methodological choices. The choices made in the BELHES were highly influenced by the limited budget. The first choice was to organize the BELHES as a second stage of the BHIS. This approach had two important advantages: 1) costs were reduced because no budget was required for sample selection and recruitment of the participants, 2) the questionnaire could be shortened because information was already collected in the HIS. A second choice, related to the first one, was that examinations were done during a home visit. Home visits may have had a positive impact on the participation rate, because they require less effort from the participant than a visit to an examination site. Alternatives to home visits are inviting participants to come to a site within existing health care properties, such as a hospital, a health center or a general practitioner’s office [31], and the use of a mobile examination site, for instance a bus equipped for examinations [6, 32]. The latter options, however, are less feasible in a setting were a HES is organized as a follow up of a HIS at the people’s home. The second stage approach, however, had some disadvantages. As the BELHES sample depended on the BHIS sample, areas where it was difficult to find BHIS interviewers were not or less covered by the BELHES. Another problem was the time delay between the day of the BHIS interview and the day of the examination. This time delay should be kept to a minimum. The aim was to have the health examination done within 3 months after BHIS participation, but in some cases it was not possible to make a timely appointment.

Another choice was to recruit participants from all over the country, except for a small area in Belgium where people speak German. This area represents only 0,6% of the Belgian population and it was felt that the extra logistical burden associated with the organization of the fieldwork in an extra language group did not justify the added value of including them.

A final choice was to limit the number of measurements in favor of EHES core measurements, which focus on cardiovascular risk factors. The decision to go for a limited number of measurements was also justified for another reason: EHES recommendations advise countries with little HES experience not to adopt too many extra measurements when setting up a first HES [33].

The EHES recommendations were followed to a great extent, although some compromises had to be made. For example, EHES recommends to have a sample size of at least 4000 participants for a national HES in order to get sufficiently precise estimates [34]. Unfortunately, the target sample size for the first BELHES was much smaller. It is well acknowledged that this smaller sample size only allows making broad estimates of the indicators of interest. Furthermore, the age range of the BELHES target group (adults aged 18 years and older, with no upper age limit) was wider than the age range proposed by EHES (25 to 64 year old). The wider age range in the BELHES ensured that even in the case of a low BELHES participation rate sufficient BHIS 2018 participants would be available to obtain the required BELHES sample size. The inclusion of more older people could have resulted into more bias. Indeed, a study demonstrated that among HES participants aged 65 years and older, there was an underrepresentation of people in residential care, from disadvantaged neighborhoods, with a lower education level, with a foreign citizenship, or with a lower health-related quality of life [35].

Selection bias is a general concern in population surveys. In case of a HES the collection of biological samples (blood and urine) may reduce the participation rate because an examination is more invasive than a simple questionnaire. However, having a free check of cholesterolaemia and glycaemia could also work as an incentive for participation. In order to avoid an impact on the BHIS participation, BELHES participation among eligible BHIS participants was optional. As a result of this choice, selection bias may have occurred at two occasions: first, among people who were invited for the BHIS, second, among BHIS participants who were invited for the BELHES.

A specific concern of the BELHES sample is the underrepresentation of low educated people. During the recruitment stage of the BHIS 2018, non-participating-households were substituted by households with similar characteristics in terms of statistical sector, household size and age of the reference person (head of household). To match the population structure of the BELHES sample to the distribution of the general Belgian population in terms of gender, age and province, post stratification weights were calculated by making use of the Belgian NR. Unfortunately, educational attainment could not be taken into account to calculate the survey weights, because this information was not available in the NR. This is a concern, because it is well known that a higher socio-economic status positively affects health survey participation [36, 37]. The second stage recruitment of the BELHES seems to have reinforced the underrepresentation of low educated people, who were already underrepresented in the BHIS. The underrepresentation of low educated people in the BELHES sample should be taken into account when interpreting the BELHES results.

Despite the limitations, it is felt that the BELHES provided very valuable results. The BELHES was also an important learning process, which will be very useful for future national HESs that will be organized in Belgium.

## Conclusion

For the first time in Belgium, a HES was successfully organized as a second stage of a national HIS. The BELHES contains objective information on the prevalence of chronic disease risk factors such as overweight and obesity, hypertension, diabetes and hypercholesterolaemia. Despite restrictions imposed by limited resources, the EHES recommendations were followed to a great extent. This offers the opportunity to compare the results with those of other recent national surveys in Europe that used the same EHES methodology. Furthermore, the results of this survey will be valuable to underpin future health policies in Belgium.

## Supplementary information

**Additional file 1.** Modules included in the BHIS 2018.

## Data Availability

Microdata of the BELHES can be obtained from the authors after approval by the Belgian data protection authority.

## Abbreviations

BELHES: Belgian Health Examination Survey
BHIS: Belgian Health Interview Survey
EHES: European Health Examination Survey
EU: European Union
HIS: Health Interview Survey
HES: Health Examination Survey
NHANES: National Health and Nutrition Examination Survey
UK: United Kingdom
DEGS: German Health Interview and Examination Survey for Adults
Esteban: Etude de santé sur l’environnement, la biosurveillance, l’activité physique et la nutrition
EHES-Lux: European Health Examination Survey in Luxemburg
CAPI: Computer assisted personal interviewing
HbA1c: Glycated haemoglobin
PAHs: Polyaromatic hydrocarbons
SHARE: Survey of Health, Ageing and Retirement
BMI: Body Mass Index
DNA: Deoxyribonucleic acid
WHO: World Health Organization
Statbel: Statistics Belgium
ID: Identification
NESCAV: Nutrition, Environment and Cardiovascular health survey
NR: National Register

## References

[1] World Health Organization. Noncommunicable diseases report 2018. Geneva: World Health Organ; 2018. p. 223.

[2] Weisz G (2011). Epidemiology and health care reform. Am J Public Health.

[3] Tolonen H, Koponen P, Al-kerwi A, Capkova N, Giampaoli S, Mindell J (2018). European health examination surveys - a tool for collecting objective information about the health of the population. Arch Public Health.

[4] Paalanen L, Härkänen T, Tolonen H (2019). Protocol of a research project “projections of the burden of disease and disability in Finland - health policy prospects” using cross-sectional health surveys and register-based follow-up. BMJ Open.

[5] Oyebode O, Mindell JS. A review of the use of health examination data from the health survey for England in government policy development and implementation. Vol. 72, Archives of Public Health: BioMed Central Ltd; 2014. 10.1186/2049-3258-72-24 PMC4128608 25114791

[6] Scheidt-Nave C, Kamtsiuris P, Göwald A, Hölling H, Lange M, Busch MA (2012). German health interview and examination survey for adults (DEGS) - Design, objectives and implementation of the first data collection wave. BMC Public Health.

[7] Rijksinstituut voor Volksgezondheid en Milieu (RIVM) (2011). Nederland de Maat Genomen, 2009–2010. Monitoring van risicofactoren in de algemene bevolking.

[8] Balicco A, Oleko A, Szego E, Boschat L, Deschamps V, Saoudi A (2017). Protocole Esteban : une Étude transversale de santé sur l’environnement, la biosurveillance, l’activité physique et la nutrition (2014–2016). Toxicol Anal Clin.

[9] Bocquet V, Barré J, Couffignal S, D’Incau M, Delagardelle C, Michel G (2018). Study design and characteristics of the Luxembourg European Health Examination Survey (EHES-LUX). BMC Public Health.

[10] Tolonen H, 2nd (2016). EHES Manual. Part A. Planning and preparation of the survey.

[11] Demarest S, Van der Heyden J, Charafeddine R, Drieskens S, Gisle L, Tafforeau J (2013). Methodological basics and evolution of the Belgian health interview survey 1997–2008. Arch Public Health.

[12] Mindell JS, Giampaoli S, Goesswald A, Kamtsiuris P, Mann C, Männistö S (2015). Sample selection, recruitment and participation rates in health examination surveys in Europe - experience from seven national surveys. BMC Med Res Methodol.

[13] Tolonen H, Mäki-Opas J, Mindell JS, Trichopoulou A, Naska A, Männistö S (2017). Standardization of physical measurements in European health examination surveys - experiences from the site visits. Eur J Pub Health.

[14] Romero-Ortuno R, O’Shea D, Kenny RA (2011). The SHARE frailty instrument for primary care predicts incident disability in a European population-based sample. Qual Prim Care.

[15] Romero-Ortuno R (2013). The frailty instrument for primary care of the survey of health, ageing and retirement in Europe predicts mortality similarly to a frailty index based on comprehensive geriatric assessment. Geriatr Gerontol Int.

[16] Stiffler MPHKA, Wilber MPHST, Frey J, McQuown CM, Poland S (2016). Frailty defined by the SHARE Frailty Instrument and adverse outcomes after an ED visit.

[17] Xue QL (2011). The frailty syndrome: definition and natural history. Clin Geriatr Med.

[18] Drieskens S, Demarest S, Bel S, De Ridder K, Tafforeau J (2018). Correction of self-reported BMI based on objective measurements: a Belgian experience. Arch Public Health.

[19] Carmienke S, Freitag MH, Pischon T, Schlattmann P, Fankhaenel T, Goebel H (2013). General and abdominal obesity parameters and their combination in relation to mortality: a systematic review and meta-regression analysis. Eur J Clin Nutr.

[20] de Hollander EL, Bemelmans WJ, Boshuizen HC, Friedrich N, Wallaschofski H, Guallar-castillón P (2012). The association between waist circumference and risk of mortality considering body mass index in 65- to 74-year-olds: a meta-analysis of 29 cohorts involving more than 58 000 elderly persons. Int J Epidemiol.

[21] Kodama S, Horikawa C, Fujihara K, Heianza Y, Hirasawa R, Yachi Y (2012). Comparisons of the strength of associations with future type 2 diabetes risk among anthropometric obesity indicators, including waist-to-height ratio: a meta-analysis. Am J Epidemiol.

[22] WHO. Waist circumference and waist-hip ratio: report of a WHO expert consultation. Geneva; 2008. Available from: www.who.int. Accessed 6 Dec 2019.

[23] Celis-Morales CA, Welsh P, Lyall DM, Steell L, Petermann F, Anderson J (2018). Associations of grip strength with cardiovascular, respiratory, and cancer outcomes and all cause mortality: prospective cohort study of half a million UK biobank participants. BMJ..

[24] Bohannon RW (2015). Muscle strength: clinical and prognostic value of hand-grip dynamometry. Curr Opin Clin Nutr Metab Care.

[25] Roberts HC, Denison HJ, Martin HJ, Patel HP, Syddall H, Cooper C, et al. A review of the measurement of grip strength in clinical and epidemiological studies: towards a standardised approach, Age Ageing. 2011;40:423–9. 10.1093/ageing/afr051 21624928

[26] WHO. Global action plan for the prevention and control of NCDs 2013-2020: WHO; 2015.

[27] Dunn JT, Crutchfield HE, Gutekunst R, Dunn AD (1993). Two simple methods for measuring iodine in urine. Thyroid..

[28] Jarvis MJ, Feyerabend C (2015). Recent trends in children’s exposure to second-hand smoke in England: cotinine evidence from the health survey for England. Addiction..

[29] Paci E, Pigini D, Bauleo L, Ancona C, Forastiere F, Tranfo G (2018). Urinary cotinine concentration and self-reported smoking status in 1075 subjects living in central Italy. Int J Environ Res Public Health.

[30] Alkerwi A, Guillaume M, Zannad F, Laufs U, Lair ML (2010). Nutrition, environment and cardiovascular health (NESCAV): protocol of an inter-regional cross-sectional study. BMC Public Health.

[31] Sylvie S. Contribution to the epidemiology of migraine and associated cardiovacular risk factors: UCLiège; 2018.

[32] Knekt P, Rissanen H, Järvinen R, Heliövaara M (2017). Cohort profile: the Finnish mobile clinic health surveys FMC, FMCF and MFS. Int J Epidemiol.

[33] Kuulasmaa K, Tolonen H, Koponen P, Kilpeläinen K, Avdicová M, Broda G (2012). An overview of the European health examination survey pilot joint action.

[34] Kuulasmaa K, Tolonen H. What is EHES and why is it needed. Available from: http://www.ehes.info. Accessed 6 Dec 2019.

[35] Gaertner B, Seitz I, Fuchs J, Busch MA, Holzhausen M, Martus P (2016). Baseline participation in a health examination survey of the population 65 years and older: who is missed and why?. BMC Geriatr.

[36] Demarest S, Van Der Heyden J, Charafeddine R, Tafforeau J, Van Oyen H, Van Hal G (2013). Socio-economic differences in participation of households in a Belgian national health survey. Eur J Pub Health.

[37] Boshuizen HC, Viet AL, Picavet HSJ, Botterweck A, van Loon AJM (2006). Non-response in a survey of cardiovascular risk factors in the Dutch population: determinants and resulting biases. Public Health.

