# Supplementary material for: The Belgian health examination survey: objectives, design and methods
Source: Arch Public Health. 2020 Jun 3;78:50. doi: 10.1186/s13690-020-00428-9 (PMC7268416; doi:10.1186/s13690-020-00428-9)
Supplement: Supplementary file 1 — Additional file 1. Modules included in the BHIS 2018. [file 13690_2020_428_MOESM1_ESM.docx]

**Additional file 1**

Modules included in the BHIS 2018

| Health and quality of life | Minimum European Health Module  Chronic diseases  Long-term limitations  Health related quality of life  Bodily pain  Frailty  Absence from work  Oral health |
| --- | --- |
| Mental health and well-being | Stress and well-being (WB)  Energy/vitality (VT)  Life satisfaction (VT)  Eating disorders  Anxiety disorders  Depressive disorders  Suicidal behaviours  Self-reported depression and treatment  Use of psycho-pharmaceuticals  Child & adolescent mental health |
| Health behaviours and lifestyle | Alcohol consumption  Tobacco smoking  E-cigarettes use  Use of illicit drugs  Gambling  Physical activity  Nutritional status  Nutritional habits  Sexual health |
| Health and society | Lodging conditions  Health and environment  Exposure to tobacco smoke / e-vapours  Accidents and injuries  Violence  Social health  Informal care |
| Preventive knowledge and practices | Cancer screening  Vaccination  Cardiovascular and diabetes risk screening  Knowledge and attitudes about HIV/AIDS  Health literacy |
| Health care consumption | Contacts with the general practitioner  Contacts with the specialist  Contacts with the dentist  Hospitalisation  Contact with paramedics  Contacts with non-conventional medicine  Contacts with home care services  Accessibility to health care  Patient experiences  Use of medicines |
